# Does tDCS Enhance Complex Motor Skill Acquisition? Evidence from a Golf-Putting Task

**DOI:** 10.3390/s25144297

**Published:** 2025-07-10

**Authors:** Virginia Lopez-Alonso, Gabriel López-Bermúdez, Jeffrey Cayaban Pagaduan, Jose Andrés Sánchez-Molina

**Affiliations:** 1 Faculty of Sciences of Sport and Physical Education, Department of Physical Education, University of A Coruña, 15179 A Coruña, Spain; jose.andres.sanchez.molina@udc.es; 2 MOBhE Group, Center of Higher Education Alberta Giménez (CESAG), Comillas Pontifical University, 07013 Palma, Spain; glopezbermudez@gmail.com; 3 CPR Plurilingüe Peñarredonda, 15008 A Coruña, Spain; 4 Brain and Mental Health Hub, Melbourne School of Psychological Sciences, Parkville, VIC 3010, Australia; jeffrey.pagaduan@unimelb.edu.au

**Keywords:** motor learning, transcranial direct current stimulation (tDCS), sport, motor cortex (M1), prefrontal cortex (PFC), golf-putting

## Abstract

**Highlights:**

**What are the main findings?**

- Repeated practice of a complex motor skill (golf-putting) led to significant performance improvements in novice individuals, regardless of the transcranial direct current stimulation (tDCS) condition.
- tDCS over the motor and prefrontal cortex did not enhance performance in the learning of a complex motor skill (golf-putting) among novice individuals.

**What is the implication of the main finding?**

- These findings suggest that motor practice alone can drive learning of complex motor skills in novices, and the role of tDCS may depend on task complexity and individual variability.
- This study contributes to a better understanding of how non-invasive brain stimulation interacts with full-body motor tasks, offering valuable insights for future sports neuroscience research.

**Abstract:**

Transcranial direct current stimulation (tDCS) modulates cortical excitability, thus inducing improvements in motor learning of simple tasks. In this study, we aimed to evaluate the effect of different tDCS conditions—anodal stimulation over the motor cortex (M1), anodal and cathodal stimulation over the prefrontal cortex (PFC), and sham—on the online and offline learning of a complex accuracy task (golf-putting) in novice golfers. Methods: A total of 40 young, healthy subjects (24 men, 16 women) without previous golf experience were randomly distributed in four groups receiving sham, anodal M1, anodal PFC or cathodal PFC tDCS. All subjects participated in two consecutive sessions. In the first session, they performed 15 blocks of 10 golf-putting along with tDCS stimulation. After 24 h, they performed the same task without tDCS. Results: Repeated measures ANOVA revealed a significant improvement in performance during the two consecutive golf-putting sessions regardless of the site and the stimulation conditions. Conclusion: Our findings suggest that tDCS over M1 or PFC does not confer additional benefits in the acquisition of complex, full-body motor skills such as golf-putting.

## 1. Introduction

Transcranial direct current stimulation (tDCS) is a non-invasive brain-stimulation technique able to modulate cortical excitability, and thus cortical function, by applying a weak electrical current induced through two scalp electrodes placed over target cortical areas [1,2]. tDCS induces variations on the resting membrane potential and, depending on the polarity of the stimulation, it can increase (anodal) or decrease (cathodal) cortical excitability [2].

Several studies have highlighted tDCS as a modulator of cognitive and motor behaviour. Consequently, the use of tDCS to enhance cognitive and motor functions has enjoyed a massive increase in popularity [3,4,5,6]. In the sport science field, studies have been mainly focused on the impact of the tDCS in power and strength tasks [7,8,9,10]. For instance, Cogimanian et al. (2007) demonstrated reduction in muscle fatigue after anodal tDCS over motor areas [11]. However, other studies have not reported positive effects of tDCS on muscle fatigue and strength [12,13]. In a more recent study, Alix-Fages et al. (2020) found an increase in training volume and a lower loss of movement velocity after anodal tDCS applied before a resistance training session [14]. Despite an absence of sufficient scientific evidence, many companies have begun to market tDCS devices under the “promise” to improve sports performance and even motor skill learning [15,16].

In this context, several studies have reported that tDCS over motor cortex (M1) and other brain areas can improve motor learning. However, the majority of the tasks evaluated consist of simple lab-based motor learning paradigms [13], such as implicit- and explicit-sequence learning tasks [17], visuomotor learning [18] or accurate motor performance tasks [19] (for a review see [20,21]). To date, a few studies have reported the effect of tDCS on sport skills that require accuracy and involve full body movement. For example, Zhu et al. (2015) [22] reported improved performance during golf-putting when cathodal tDCS was applied over the left dorsolateral prefrontal cortex (PFC). However, their protocol involved a dual-task condition on the second day, where participants completed a verbal working memory task between a retention test and a retest. While no significant differences were observed in the initial retention test, the stimulation group outperformed sham during the multitask condition, suggesting that cathodal tDCS may enhance implicit motor performance specifically under cognitive load. On the other hand, two studies reported no overall effect of tDCS on golf-putting performance following stimulation of M1, PFC, or visual cortices (Oz). Harris et al. (2019) applied unilateral anodal tDCS over M1, PFC, and Oz and found no performance benefits across any condition [23]. Similarly, Parma et al. (2020) applied bihemispheric stimulation over M1 and also reported no overall group differences [24]. However, Parma et al. observed a slight improvement in club acceleration in those subjects with a worse performance in the baseline, suggesting that individuals with poorer initial motor skills may respond more positively to tDCS. It is interesting to note that both studies applied tDCS prior to the task, rather than during its execution. Additionally, Harris et al. (2019) [23] included only 10 putts under low-pressure and 10 under high-pressure conditions, which reflect isolated performance snapshots rather than a learning trajectory. The interaction between baseline ability and stimulation efficacy is consistent with previous findings in full body motor tasks, such as dart throwing or a dance video game [25,26]. Furthermore, in a recent study, Moreira et al. [27] documented no difference in shooting accuracy between atDCS at PFC and sham among female professional basketball athletes. Furthermore, Molero-Chamizo et al. (2018) [28] suggest that tDCS applied just before a go/no-go simple reaction time task has more effects on performance compared to stimulation applied 30 or 60 min before [29]. Similarly, a recent study evaluating motor adaptation found selective enhancement of learning only when cerebellar tDCS was applied simultaneously with movement [30]. The inconsistent findings on tDCS and sports skills may be attributed to biological [28,31] and mechanical factors [29,30], highlighting the need for further research. These discrepancies from the aforementioned tDCS studies using complex tasks underscore the importance of (i) timing relative to practice (online vs. offline), (ii) stimulation montage, and (iii) stimulation polarity when interpreting tDCS outcomes. The present study incorporates methodological strengths aimed at addressing these factors. First, we applied tDCS during task execution, which allows for the assessment of its immediate effects on motor performance—an approach less commonly explored compared to pre-task stimulation protocols. Second, we targeted two functionally distinct cortical areas, M1 and PFC, to examine both motor and cognitive contributions to skill learning. Third, we included both anodal and cathodal stimulation conditions over the PFC, enabling a more comprehensive analysis of polarity-specific effects. Together, these design elements provide a more detailed investigation of how tDCS interacts with the acquisition of high-precision, full-body motor skills such as golf-putting.

Therefore, the main objective of the present study was to evaluate the effect of tDCS applied during task performance on both online and offline learning of the golf-putting skill. Although motor skills are typically acquired over extended periods of deliberate practice—often requiring a minimum of ten years to reach expert-level performance in sports [32]—rapid improvements can be observed in novices following a single practice session [33]. Performance gains achieved within a single session are referred to as online learning [19], whereas gains observed at a delayed retest—over several hours or days—without further practice are defined as offline learning [34]. Previous evidence suggests that tDCS over M1 may enhance both online learning and post-practice consolidation following sleep [35]. In the current study, we compared motor performance in a golf-putting task during a practice session with and without tDCS and assessed its effects in a second session conducted 24 h later.

Additionally, we examined the effects of tDCS over two cortical areas involved in motor skill acquisition—M1 and PFC—by assigning participants to separate groups for each stimulation condition to explore the neural processes underlying early improvements in golf-putting performance. While M1 was stimulated using anodal tDCS, the PFC was stimulated with both anodal and cathodal tDCS in different sessions. M1 is associated with implicit processes during the initial stages of motor learning [36,37], whereas the PFC has been linked to cognitive strategies and the trial-and-error approach [38,39]. The rationale for applying cathodal tDCS over the PFC is based on the findings of Zhu et al. (2015) [22], which showed that inhibiting the left PFC during a golf-putting task enhanced performance by promoting implicit motor learning. Their findings, supported by additional evidence [40,41], suggest that reducing prefrontal activity facilitates automatic motor execution and minimizes the interference of conscious control, especially in complex motor tasks. Anodal PFC stimulation, in contrast, may facilitate strategic exploration in novices who are still searching for an effective movement pattern [23]. Examining both polarities allows us to test different hypotheses about cognitive involvement during the initial stages of golf-putting learning.

## 2. Materials and Methods

### 2.1. Subjects

A total of 40 young, healthy subjects (24 men, 16 women), right-handed with no golf experience, volunteered to participate in this study. They were randomly distributed in 4 groups receiving different tDCS stimulation; sham (n = 10, 6 men, age = 21.3, SD = 1.8), anodal M1 (n = 10, 6 men, mean age = 21.5, SD = 1.8), anodal PFC (n = 10, 6 men, mean age = 20.5, SD = 1.3) and cathodal PFC (n = 10, 6 men, mean age = 22.8, SD = 3.3). The Local Ethics Committee of the University of La Coruña approved the experimental protocol, in accordance with the declaration of Helsinki. All of them submitted an informed consent and were screened for contraindications to TMS and tDCS.

### 2.2. Procedure

This study employed a randomized, single-blind, sham-controlled parallel study design. Participants visited for two occasions, separated by 24 h between 0800–1600 h at a facility in a university. Experimentation occurred at similar times to avoid diurnal effects. In the first session, a baseline block of 10 throws was performed. This was followed by 15 blocks of 10 golf-putts performed concurrently with tDCS stimulation (according to group allocation). The second session consisted of 15 blocks of 10 throws without stimulation.

### 2.3. Golf-Putting Task

The golf-putting task consists of putt standard white golf balls to a standard dimensions target hole on an artificial grass surface that was even and level with a standard golf putter. The distance to the target hole was 2.20 m. The following five instructions were given to all participants: (1) grab the club with one hand above the other; (2) place the ball in the middle of both feet; (3) knees at semi flexion with the body above the ball; (4) pendulum-like motion of the arms and golf club, avoiding wrist movement; and (5) velocity of the club determines speed of the ball. A monetary reward (50€) was offered to the best performer to increase the motivation of the participants.

Golf data were acquired through a Logitech WebCam pro 9000 (sampling frequency 25 Hz) placed over the hole, recording all the throws. Tracker Video Analysis Tool was used to calculate distances between the ball and the hole. For the golf-putting task, error was measured as the distance from the hole to the final position of the ball after the throw. When the ball ended out of WebCam range, error was assigned as the maximum error value (148 cm) [24].

### 2.4. tDCS

tDCS was delivered at 1.5 mA for 20 min through a pair of saline-soaked sponge surface electrodes (35 cm^2^) connected to a DC stimulator (neuroConn, Neurocare, Germany). For M1 stimulation, an active electrode (anode) was placed over the hotspot of the left M1 (as determined by transcranial magnetic stimulation, TMS). For PFC stimulation, the active electrode—either anode (for anodal stimulation) or cathode (for cathodal stimulation)—was placed over the left dorsolateral PFC area (F3) in accordance with the 10–20 international system for EEG electrode placement. The reference electrode, in all cases, was placed over the contralateral supraorbital region. The current was faded in and faded out 8 s each. For sham stimulation group (sham), the placement of the electrodes was the same as for the anodal M1 (aM1) group although current was applied only for 30 s.

### 2.5. Statistical Analysis

Data are presented as mean ± SD. A Shapiro–Wilk test was applied to explore the normality of the data. One-way ANOVA was used to determine baseline difference in mean absolute error (eb0) and successful putts (those that finished inside the hole). We also conducted a two-way repeated measures ANOVA with Bonferroni post hoc to examine any difference in successful putts. When the assumption of sphericity was violated, as assessed by Mauchly’s test, the Greenhouse–Geisser correction was applied to adjust the degrees of freedom for within-subject effects.

We also employed this method to identify any difference in offline learning, wherein we used the values from the last block of the first session and first block of the second session for comparison. Analyses were carried out using SPSS 29.0.2.0 (20) (Chicago, IL, USA) with significance set at 0.05 alpha.

## 3. Results

Normality was assessed using the Shapiro–Wilk test. Most variables met the normality assumption in all groups, with only minor deviations observed in isolated cases.

One-way ANOVA revealed no differences between groups in the baseline block, neither for error nor successful putts.

**Figure 1:**
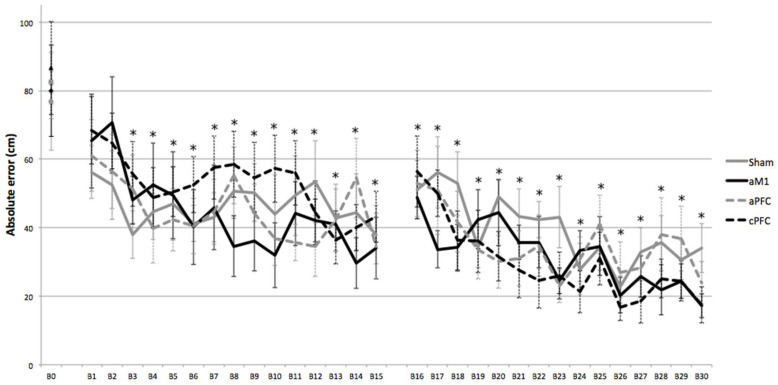
Effects of tDCS on absolute error: Evolution of absolute errors for sham, anodal motor cortex (aM1), anodal prefrontal cortex (aPFC) and cathodal prefrontal cortex (cPFC) groups along blocks (from baseline (B0) to last block of session 2 (B30)). B1 to B15 represent the first session (with stimulation), and B16 to B30 represent the second session (without stimulation). Error bars represent standard error. Asterisks indicate significant differences between the absolute error at that block and the baseline absolute error.

Two-way repeated measures ANOVA absolute errors in session 1 revealed that there was an effect of BLOCK (F = 8.616; *p* < 0.001; η^2^_p_ = 0.193). There was no effect of GROUP in putting error (F = 0.33, *p* = 0.803, η^2^_p_ = 0.027). There was also no significant interaction between BLOCK*GROUP (F = 0.77, *p* = 0.724, η^2^_p_ = 0.061). Post hoc analysis showed significant differences between baseline block and all the blocks from block 3 to block 15 (*p* < 0.01) (Figure 1).

**Figure 2:**
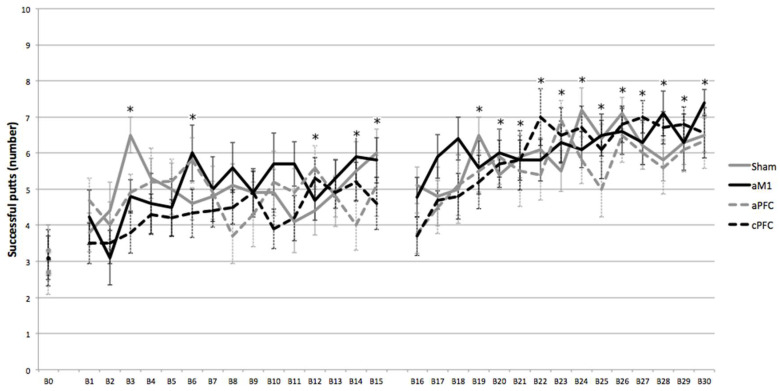
Effects of tDCS on successful putts: Evolution of successful putts for sham, anodal motor cortex (aM1), anodal prefrontal cortex (aPFC) and cathodal prefrontal cortex (cPFC) groups along blocks (from baseline (B0) to last block of session 2 (B30)). B1 to B15 represent the first session (with stimulation), and B16 to B30 represent the second session (without stimulation). Error bars represent standard error. Asterisks indicate significant differences between the successful putts at that block and the baseline successful putts.

ANOVArm for the number of successful putts per block in session 1 revealed an effect of BLOCK (F = 4.17, *p* < 0.001, η^2^_p_ = 0.112) but no effect of GROUP (F = 0.42, *p* = 0.739, η^2^_p_ = 0.037) or BLOCK*GROUP interaction (F = 1.19, *p* = 0.244, η^2^_p_ = 0.097). Post hoc analysis showed significant differences (*p* < 0.05) between baseline and blocks 3, 6, 12, 14 and 15 (Figure 2).

ANOVArm for the averaged absolute errors in session 2 revealed that there was an effect of BLOCK (F = 7.610, *p* < 0.001, η^2^_p_ = 0.175) but no effect of GROUP (F = 0.923, *p* = 0.439, η^2^_p_ = 0.071) or BLOCK*GROUP interaction (F = 0.835, *p* = 0.761, η^2^_p_ = 0.065) (Figure 1).

ANOVArm for the number of successful putts per block in session 2 revealed an effect of BLOCK (F = 4.400, *p* < 0.001, η^2^_p_ = 0.124) but no effect of GROUP (F = 0.483, *p* = 0.697, η^2^_p_ = 0.045) or BLOCK*GROUP interaction (F = 0.846, *p* = 0.742, η^2^_p_ = 0.076) (Figure 2).

ANOVArm between the last block of session 1 and first block of session 2 for averaged absolute errors revealed an effect of BLOCK (F = 7.556, *p* = 0.009, η^2^_p_ = 0.173) but no effect of GROUP (F = 0.261, *p* = 0.853, η^2^_p_ = 0.021) or BLOCK*GROUP interaction (F = 0.050, *p* = 0.985, η^2^_p_ = 0.004).

## 4. Discussion

This study aimed to examine the effects of tDCS on both online and offline learning of a golf-putting skill. Results revealed no significant differences in tDCS compared to sham in either an online or offline golf-putting task. Furthermore, no significant differences were observed between stimulation of the primary motor cortex and the prefrontal cortex, nor between anodal and cathodal stimulation of the prefrontal cortex.

The number of repetitions of golf-putting conducted in the current study was effective to improve both the online and offline learning in our subjects. However, the use of atDCS or ctDCS in combination with the motor practice failed to enhance these learning processes. Based on previous studies of tDCS influencing motor tasks performance [17,19,22], we initially expected an effect of tDCS in golf-putting. This hypothesis was also supported by the fact that corticospinal plasticity is considered responsible for mastering motor skills involved in any high-performance sport [10]. In addition, one similar study evidenced enhanced golf-putting performance during practice on the first day with ctDCS over PFC [22]. However, no group differences were found in the initial retention test conducted the following day. This suggests that the effects of stimulation may be transient or task-dependent. In contrast, our study did not find any significant between-group differences at any point, neither during practice nor in the retention session, despite a similar stimulation intensity and task. Unlike most comparable studies, our protocol included a financial reward for the best performer. While this likely enhanced overall motivation and participant engagement, it may have also elevated baseline effort levels across all conditions, reducing performance variability and potentially masking subtle group differences. Moreover, while previous research has shown that tDCS can facilitate learning in whole-body balance tasks [42], golf-putting is a precision-demanding skill that relies on distinct control strategies and neural circuits [43].

Furthermore, although our results showed no significant effects of tDCS across groups, we acknowledge that individual differences in baseline motor skill or cognitive capacity could moderate responsiveness to stimulation. For example, Parma et al. (2020) [24] found no overall group effect of tDCS on golf-putting performance but reported a selective improvement in club acceleration among participants with lower baseline proficiency. While this nuance highlights the potential moderating role of initial skill level, the overall pattern of null group-level effects observed in both Parma et al. (2020) [24] and in our study remains consistent. Similarly, Harris et al. (2019) [23] also found no effect of anodal tDCS over motor, frontal, or visual cortices on golf-putting performance. Taken together, these findings imply that, under certain conditions, tDCS may not reliably enhance performance in precision-based motor tasks such as golf-putting. Although we did collect baseline performance data, we chose not to stratify participants by initial skill due to the relatively small sample size, which would have substantially limited the power of subgroup comparisons. Moreover, while baseline-dependent stimulation effects have been reported in other contexts (e.g., dart throwing or dance-game tasks [25,26]), further research is needed to determine the influence of individual variability on DCS responsiveness in golf-specific motor learning. Additionally, traits such as working memory capacity (particularly relevant when stimulating the DLPFC) may also interact with task demands and stimulation effects. Our results also align with studies that reported a lack of effect of tDCS in the performance and learning of different motor tasks [28,44,45]. Therefore, the non-effect of tDCS in our study may be accounted from various factors that warrant further investigation.

Various factors such as inter-individual variability, intensity of stimulation, and area of stimulation may account for the non-effect of atDCS/ctDCS in this study. It has been demonstrated that not all subjects show the expected modulation in cortical excitability in response to tDCS [46,47,48,49]. Although the neurophysiological modulation induced by the tDCS may not be linearly correlated with the ability for motor learning [50,51], a lack of effect of tDCS in some subjects could influence the negative results. The intensity of stimulation used in our experiment was 1.5 mV, in line with previous studies that observed a higher effect on motor learning at that intensity [22]. A task-dependent effect of tDCS could explain our lack of improvement in comparison with those studies and in line with studies reporting no effects of tDCS on golf-putting learning [23,24]. The concurrent combination of anodal stimulation and motor task, both increasing excitability, may trigger non-additive mechanisms, impairing neuroplasticity [52,53]. Therefore, it is also unlikely that the use of higher intensities (i.e., 2 mV) could be more efficient since it has shown a reverse effect in cortical excitability [54]. However, we cannot discard that lower intensities of stimulation than the ones used in our study would lead to a different result. Nevertheless, the relationship between this parameter of stimulation and its effects depending on the task is still a topic of debate.

In the present study, we compared the motor performance during a practical session of golf-putting, with and without atDCS/ctDCS, and its effects in a second session of practice carried out 24 h later. We stimulated PFC and M1, which have been demonstrated to play a role in motor learning [55]. Specifically, M1 has been associated with the implicit process present in the early phases of motor learning acquisition [36,37], while PFC is linked to the cognitive strategies of a trial-and-error approach [38,39]. Although motor skills are typically slow-paced and learned over multiple days of practice (at least 10 years of practice are needed for an expert performance in sport) [32], one session is enough to observe rapid improvements in inexperienced subjects [33]. Performance improvements obtained as the result of shorter time periods of practice, such as within a single session, are referred to as online learning [19]. In contrast, performance improvements at re-test in the absence of additional practice (over several hours or days) are referred as offline learning [34]. The atDCS over M1 seems to improve not only online learning but also consolidation after overnight sleep [35].

However, atDCS may influence brain networks beyond the stimulated areas [56]. During a full body movement activity such as golf-putting, this “unintentional stimulation” could interfere with other brain functions relevant for motor skill acquisitions. Therefore, the stimulation over one specific area may not be the optimal strategy to influence the cortical networks underlying the learning of an accurate motor task such as the one used in our study.

In this study, it may be possible that the non-differences between atDCS and sham may be attributed to the ramp-up and down stimulus, inducing cortical excitability or placebo effects. Future studies should warrant tDCS configurations to avoid bias or unaware observations.

We must point out that in the current study, we explored the effect of tDCS on motor learning in novice practitioners. It is known that several differences exist between novice and expert golfers, from neuroanatomical features [57] to the use of cognitive strategies such as the focus of attention [58,59] or feedback [60,61]. In addition, novice subjects showed heterogeneity in behaviour and difficulties when filtering out irrelevant information [62]. Although we provided standardized verbal instructions to all subjects [63] and tried to keep them motivated and engaged in the practice (by using a reward) to minimize possible sources of intra- and inter-subject variability, it is possible that those factors could mask beneficial effects of tDCS.

One methodological limitation of the present study concerns the lack of precise timing data for the duration of individual practice sessions. Although the stimulation protocol was fixed at 20 min, we did not systematically record the exact time each participant took to complete the motor task. Researcher observations suggested that all participants finished the practice phase within approximately ±3 min of the stimulation window, ensuring general temporal overlap between tDCS and task execution; nevertheless, small variations may have introduced minor inconsistencies in stimulation-task alignment. Additionally, the relatively small sample size limits the generalizability of the findings and reduces statistical power to detect small or moderate effects. Despite these limitations, this study offers valuable insights by incorporating both motor and prefrontal stimulation targets, assessing online and offline learning, and using a realistic accuracy-based task. Future research should address these limitations by systematically recording task durations, increasing sample sizes, stratifying participants by skill level, assessing cognitive-motor profiles, and exploring dose–response relationships. Moreover, comparing online versus offline stimulation effects and replicating the protocol in skilled or sport-specialized populations—where learning dynamics and ceiling effects may differ—would further enhance understanding of tDCS effects on motor learning.

## 5. Conclusions

Non-invasive brain stimulation techniques are postulated as tools to improve performance in sports. Although we found no effects of tDCS in improving performance of golf-putting, the demands vastly differ between sports; thus, these results could not be extrapolated to other disciplines. More studies with other sport skills are needed to draw solid conclusions.

## Data Availability

The data that support the findings of this study are available from the corresponding author, VLA, upon reasonable request.

## Abbreviations

The following abbreviations are used in this manuscript:

tDCS	Transcranial direct current stimulation
M1	Motor cortex
PFC	Prefrontal cortex
TMS	Transcranial magnetic stimulation
aM1	Anodal M1
eb0	Baseline absolute error

## References

[1] Nitsche M.A., Paulus W. (2000). Excitability changes induced in the human motor cortex by weak transcranial direct current stimulation. J. Physiol..

[2] Priori A., Berardelli A., Rona S., Accornero N., Manfredi M. (1998). Polarization of the human motor cortex through the scalp. Neuroreport.

[3] Bashir S., Bamugaddam A., Alasheikh M., Alhassan T., Alhaidar S., Almutairi A.K., Alfaleh M., Al-Regaiey K., Al Zahrani S.S., Albaiji B.A. (2022). Anodal transcranial direct current stimulation (tDCS) over the primary motor cortex (M1) enhances motor response inhibition and visual recognition memory. Med. Sci. Monit. Basic Res..

[4] Nakashima S., Koeda M., Ikeda Y., Hama T., Funayama T., Akiyama T., Arakawa R., Tateno A., Suzuki H., Okubo Y. (2021). Effects of anodal transcranial direct current stimulation on implicit motor learning and language-related brain function: An fMRI study. Psychiatry Clin. Neurosci..

[5] Summers J.J., Kang N., Cauraugh J.H. (2016). Does transcranial direct current stimulation enhance cognitive and motor functions in the ageing brain? A systematic review and meta- analysis. Ageing Res. Rev..

[6] Yamamoto S., Ishii D., Ishibashi K., Kohno Y. (2022). Transcranial direct current stimulation of the dorsolateral prefrontal cortex modulates cognitive function related to motor execution during sequential task: A randomized control study. Front. Hum. Neurosci..

[7] Angius L., Mauger A.R., Hopker J., Pascual-Leone A., Santarnecchi E., Marcora S.M. (2018). Bilateral extracephalic transcranial direct current stimulation improves endurance performance in healthy individuals. Brain Stimul..

[8] Banissy M.J., Muggleton N.G. (2013). Transcranial direct current stimulation in sports training: Potential approaches. Front. Hum. Neurosci..

[9] Grosprêtre S., Grandperrin Y., Nicolier M., Gimenez P., Vidal C., Tio G., Haffen E., Bennabi D. (2021). Effect of transcranial direct current stimulation on the psychomotor, cognitive, and motor performances of power athletes. Sci. Rep..

[10] Nielsen J.B., Cohen L.G. (2008). The Olympic brain. Does corticospinal plasticity play a role in acquisition of skills required for high-performance sports?. J. Physiol..

[11] Cogiamanian F., Marceglia S., Ardolino G., Barbieri S., Priori A. (2007). Improved isometric force endurance after transcranial direct current stimulation over the human motor cortical areas. Eur. J. Neurosci..

[12] Montenegro R., Okano A., Gurgel J., Porto F., Cunha F., Massaferri R., Farinatti P. (2015). Motor cortex tDCS does not improve strength performance in healthy subjects. Mot. Rev. De Educ. Física.

[13] Tanaka S., Sandrini M., Cohen L.G. (2011). Modulation of motor learning and memory formation by non-invasive cortical stimulation of the primary motor cortex. Neuropsychol. Rehabil..

[14] Alix-Fages C., García-Ramos A., Calderón-Nadal G., Colomer-Poveda D., Romero-Arenas S., Fernández-del-Olmo M., Márquez G. (2020). Anodal transcranial direct current stimulation enhances strength training volume but not the force–velocity profile. Eur. J. Appl. Physiol..

[15] Garner C.T., Dykstra R.M., Hanson N.J., Miller M.G. (2021). Transcranial direct current stimulation with the halo sport does not improve performance on a three-minute, high intensity cycling test. Int. J. Exerc. Sci..

[16] Huang L., Deng Y., Zheng X., Liu Y. (2019). Transcranial direct current stimulation with halo sport enhances repeated sprint cycling and cognitive performance. Front. Physiol..

[17] Nitsche M.A., Schauenburg A., Lang N., Liebetanz D., Exner C., Paulus W., Tergau F. (2003). Facilitation of implicit motor learning by weak transcranial direct current stimulation of the primary motor cortex in the human. J. Cogn. Neurosci..

[18] Antal A., Nitsche M.A., Kincses T.Z., Kruse W., Hoffmann K.P., Paulus W. (2004). Facilitation of visuo-motor learning by transcranial direct current stimulation of the motor and extrastriate visual areas in humans. Eur. J. Neurosci..

[19] Reis J., Schambra H.M., Cohen L.G., Buch E.R., Fritsch B., Zarahn E., Krakauer J.W. (2009). Noninvasive cortical stimulation enhances motor skill acquisition over multiple days through an effect on consolidation. Proc. Natl. Acad. Sci. USA.

[20] Krakauer J.W., Mazzoni P. (2011). Human sensorimotor learning: Adaptation, skill, and beyond. Curr. Opin. Neurobiol..

[21] Reis J., Robertson E., Krakauer J.W., Rothwell J., Marshall L., Gerloff C., Cohen L.G. (2008). Consensus: “Can tDCS and TMS enhance motor learning and memory formation?”. Brain Stimul..

[22] Zhu F.F., Yeung A.Y., Poolton J.M., Lee T.M., Leung G.K., Masters R.S. (2015). Cathodal Transcranial Direct Current Stimulation Over Left Dorsolateral Prefrontal Cortex Area Promotes Implicit Motor Learning in a Golf Putting Task. Brain Stimul..

[23] Harris D.J., Wilson M.R., Buckingham G., Vine S.J. (2019). No effect of transcranial direct current stimulation of frontal, motor or visual cortex on performance of a self-paced visuomotor skill. Psychol. Sport. Exerc..

[24] Parma J.O., Profeta V.L.D.S., Andrade A.G.P.D., Lage G.M., Apolinário-Souza T. (2020). TDCS of the primary motor cortex: Learning the absolute dimension of a complex motor task. J. Mot. Behav..

[25] Mizuguchi N., Katayama T., Kanosue K. (2018). The effect of cerebellar transcranial direct current stimulation on a throwing task depends on individual level of task performance. Neuroscience.

[26] Suzuki K., Suzuki T., Ono Y. (2017). Effect of middletemporal tDCS stimulation on dance-game exercise performance. Trans. Jpn. Soc. Med. Biol. Eng..

[27] Moreira A., Moscaleski L., Machado D.G.D.S., Bikson M., Unal G., Bradley P.S., Cevada T., da Silva F.T.G., Baptista A.F., Morya E. (2023). Transcranial direct current stimulation during a prolonged cognitive task: The effect on cognitive and shooting performances in professional female basketball players. Ergonomics.

[28] Molero-Chamizo A., Alameda Bailén J.R., Garrido Béjar T., García López M., Jaén Rodríguez I., Gutiérrez Lérida C., Rivera-Urbina G.N. (2018). Poststimulation time interval-dependent effects of motor cortex anodal tDCS on reaction-time task performance. Cogn. Affect. Behav. Neurosci..

[29] Horvath J.C., Carter O., Forte J.D. (2016). No significant effect of transcranial direct current stimulation (tDCS) found on simple motor reaction time comparing 15 different simulation protocols. Neuropsychologia.

[30] Vergallito A., Feroldi S., Pisoni A., Romero Lauro L.J. (2022). Inter-Individual Variability in tDCS Effects: A Narrative Review on the Contribution of Stable, Variable, and Contextual Factors. Brain Sci..

[31] Weightman M., Brittain J.S., Hall A., Miall C., Jenkinson N. (2022). Timing is everything: Event-related transcranial direct current stimulation improves motor adaptation. Brain Stimul..

[32] Ericsson K.A., Lehmann A.C. (1996). Expert and exceptional performance: Evidence of maximal adaptation to task constraints. Annu. Rev. Psychol..

[33] Dayan E., Cohen L.G. (2011). Neuroplasticity subserving motor skill learning. Neuron.

[34] Doyon J., Benali H. (2005). Reorganization and plasticity in the adult brain during learning of motor skills. Curr. Opin. Neurobiol..

[35] Kim T., Kim H., Wright D.L. (2021). Improving consolidation by applying anodal transcranial direct current stimulation at primary motor cortex during repetitive practice. Neurobiol. Learn. Mem..

[36] Iezzi E., Suppa A., Conte A., Agostino R., Nardella A., Berardelli A. (2010). Theta-burst stimulation over primary motor cortex degrades early motor learning. Eur. J. Neurosci..

[37] Karni A., Meyer G., Jezzard P., Adams M.M., Turner R., Ungerleider L.G. (1995). Functional MRI evidence for adult motor cortex plasticity during motor skill learning. Nature.

[38] Marinelli L., Quartarone A., Hallett M., Frazzitta G., Ghilardi M.F. (2017). The many facets of motor learning and their relevance for Parkinson’s disease. Clin. Neurophysiol..

[39] Rivera-Urbina G.N., Molero-Chamizo A., Nitsche M.A. (2022). Discernible effects of tDCS over the primary motor and posterior parietal cortex on different stages of motor learning. Brain Struct. Funct..

[40] Hamzei F., Ritter A., Güllmar D. (2025). Implicit Motor Learning Under Anodal or Cathodal tDCS During fMRI Induces Partially Distinct Network Responses. Eur. J. Neurosci..

[41] Luft C.D.B., Zioga I., Banissy M.J., Bhattacharya J. (2017). Relaxing learned constraints through cathodal tDCS on the left dorsolateral prefrontal cortex. Sci. Rep..

[42] Kaminski E., Hoff M., Sehm B., Taubert M., Conde V., Steele C.J., Ragert P. (2013). Effect of transcranial direct current stimulation (tDCS) during complex whole body motor skill learning. Neurosci. Lett..

[43] Ungerleider L.G., Doyon J., Karni A. (2002). Imaging brain plasticity during motor skill learning. Neurobiol. Learn. Mem..

[44] Hashemirad F., Fitzgerald P.B., Zoghi M., Jaberzadeh S. (2017). Single-Session Anodal tDCS with Small-Size Stimulating Electrodes Over Frontoparietal Superficial Sites Does Not Affect Motor Sequence Learning. Front. Hum. Neurosci..

[45] Minarik T., Sauseng P., Dunne L., Berger B., Sterr A. (2015). Effects of anodal transcranial direct current stimulation on visually guided learning of grip force control. Biology.

[46] Guerra A., Lopez-Alonso V., Cheeran B., Suppa A. (2017). Solutions for managing variability in non-invasive brain stimulation studies. Neurosci. Lett..

[47] Guerra A., Lopez-Alonso V., Cheeran B., Suppa A. (2017). Variability in non-invasive brain stimulation studies: Reasons and results. Neurosci. Lett..

[48] Lopez-Alonso V., Cheeran B., Rio-Rodriguez D., Fernandez-Del-Olmo M. (2014). Inter-individual Variability in Response to Non-invasive Brain Stimulation Paradigms. Brain Stimul..

[49] Wiethoff S., Hamada M., Rothwell J.C. (2014). Variability in Response to Transcranial Direct Current Stimulation of the Motor Cortex. Brain Stimul..

[50] Li Voti P., Conte A., Suppa A., Iezzi E., Bologna M., Aniello M.S., Berardelli A. (2011). Correlation between cortical plasticity, motor learning and BDNF genotype in healthy subjects. Exp. Brain Res..

[51] Lopez-Alonso V., Cheeran B., Fernandez-del-Olmo M. (2015). Relationship Between Non-invasive Brain Stimulation-induced Plasticity and Capacity for Motor Learning. Brain Stimul..

[52] Bortoletto M., Pellicciari M.C., Rodella C., Miniussi C. (2015). The interaction with task-induced activity is more important than polarization: A tDCS study. Brain Stimul..

[53] Saucedo Marquez C.M., Zhang X., Swinnen S.P., Meesen R., Wenderoth N. (2013). Task-specific effect of transcranial direct current stimulation on motor learning. Front. Hum. Neurosci..

[54] Batsikadze G., Moliadze V., Paulus W., Kuo M.F., Nitsche M.A. (2013). Partially non-linear stimulation intensity-dependent effects of direct current stimulation on motor cortex excitability in humans. J. Physiol..

[55] Hardwick R.M., Rottschy C., Miall R.C., Eickhoff S.B. (2013). A quantitative meta-analysis and review of motor learning in the human brain. Neuroimage.

[56] Nitsche M.A., Doemkes S., Karakose T., Antal A., Liebetanz D., Lang N., Paulus W. (2007). Shaping the effects of transcranial direct current stimulation of the human motor cortex. J. Neurophysiol..

[57] Jancke L., Koeneke S., Hoppe A., Rominger C., Hanggi J. (2009). The architecture of the golfer’s brain. PLoS ONE.

[58] Kearney P. (2015). A distal focus of attention leads to superior performance on a golf putting task. Int. J. Sport. Exerc. Psychol..

[59] Wulf G., Lauterbach B., Toole T. (1999). The learning advantages of an external focus of attention in golf. Res. Q. Exerc. Sport..

[60] Ishikura T. (2008). Reduced relative frequency of knowledge of results without visual feedback in learning a golf-putting task. Percept. Mot. Skills.

[61] Keogh J.W., Hume P.A. (2012). Evidence for biomechanics and motor learnig research improving golf performance. Sports Biomech..

[62] Milton J., Solodkin A., Hlustik P., Small S.L. (2007). The mind of expert motor performance is cool and focused. Neuroimage.

[63] Munzert J., Maurer H., Reiser M. (2014). Verbal-motor attention-focusing instructions influence kinematics and performance on a golf-putting task. J. Mot. Behav..

